# Development of Re-Usable Yeast-Gellan Gum Micro-Bioreactors for Potential Application in Continuous Fermentation to Produce Bio-Ethanol

**DOI:** 10.3390/pharmaceutics3040731

**Published:** 2011-10-17

**Authors:** Sook Mun Tan, Paul Wan Sia Heng, Lai Wah Chan

**Affiliations:** Department of Pharmacy, Faculty of Science, National University of Singapore, 18 Science Drive 4, S117543, Singapore

**Keywords:** microencapsulation, water-in-oil emulsion, gellan gum, micro-bioreactors, bioethanol

## Abstract

The objectives of this study were to investigate the feasibility of encapsulating yeast cells using gellan gum by an emulsification method and to evaluate the fermentation efficiency and the reusability of the micro-bioreactors produced. It was found that yeast cells could be successfully encapsulated to form relatively spherical micro-bioreactors with high specific surface area for mass transfer. Cell viability was found to be reduced by one log reduction after the emulsification process. The ethanol yield of the micro-bioreactors was comparable to that of free yeast in the first fermentation cycle. The micro-bioreactors remained intact and could be re-used up to 10 cycles of fermentation. Despite cell breakthrough, relatively high ethanol yields were obtained, indicating that the micro-bioreactors also functioned as regenerative reservoirs of yeast.

## 1. Introduction

There is strong commercial interest in bio-ethanol production due to depleting sources of fossil fuel [1]. Yeast had been successfully immobilized for fermentation processes to produce bio-ethanol. Melzoch *et al.* [2] and Dror *et al.* [3] also reported that the microbial cells entrapped in gels demonstrated higher survival rates compared to free microbial cells. Besides serving as a barrier to harmful by-products, the encapsulant matrix provides mechanical support to the encapsulated cells. Encapsulation of yeast cells within a polymer matrix was also found to increase the osmotolerance of the encapsulated yeast cells [4]. Yeast cells had been successfully immobilized in alginate and carrageenan microspheres for fermentation processes to produce bio-ethanol. The immobilized yeast was found to be protected from environmental stress and ethanol toxicity, enabling higher fermentation efficiency compared to free yeast cells [3,5–8]. Thus, the use of encapsulated yeast is advocated to improve fermentation productivity and reduce production cost. Furthermore, micro-bioreactors of high stability could be easily recovered from the fermentation medium and re-used. Micro-bioreactors in the form of microspheres with large surface area to volume ratio are preferred to facilitate efficient mass transfer [9,10]. The emulsification method is an established method used for the production of microspheres to encapsulate drugs using different types of polymers [11–15]. However, it is unclear if this method is feasible for the encapsulation of yeast cells, which are more sensitive than most drugs. Furthermore, the use of gellan gum in cell encapsulation by the emulsification method has not been explored. Hence, the aim of this study was to investigate the feasibility of the emulsification method to encapsulate yeast cells using gellan gum. Gellan gum is a linear anionic polymer composed of repeating units of tetrasaccharide of glucose, glucuronic acid and rhamnose residues [16–18]. Gelation of gellan gum is induced at low temperature as the gellan gum forms double helix structure. Presence of cations such as calcium promotes aggregation of the helices and stabilizes the gellan gum matrix [19]. Gellan gum has been reported to form a relatively stable, acid- and enzyme-resistant gel [19], which renders it more resistant to degradation by heat, extreme pH and microorganisms. Thus, gellan gum is a promising encapsulating material for yeast cells to produce re-usable micro-bioreactors for bio-ethanol production.

## 2. Experimental Section

### 2.1. Preparation of Yeast for Microencapsulation

One g of active dry yeast (Turbo Extra Yeast, Still Spirit, New Zealand), containing approximately 1 × 10^9^ cells, was hydrated in 30 g of sterile distilled water for 10 min. The yeast suspension was then centrifuged (RA-200J, Kubota 1720, Japan) at 9838 × *g* and 4 °C for 10 min. The yeast pellet was collected and suspended in 1 mL of sterile deionized water for microencapsulation and other studies.

### 2.2. Microencapsulation of Yeast

Fifty g of 1.5%, w/w gellan gum solution was prepared by dissolving an appropriate amount of the gellan gum powder in hot deionized water. The solution was then sterilized by autoclaving at 121 °C for 15 min. The sterilized solution was allowed to cool to 40 °C. One mL of yeast suspension (1 × 10^9^ cells) was added into the sterilized 1.5%w/w gellan gum solution that was previously cooled to 40 °C. The mixture was stirred at 50 rpm in a beaker for 5 min at 40 °C using a mechanical stirrer (PX-OS 2000, Polymix, Germany). It was then dispersed in 75.0 g of iso-octane (Analytical Grade, Merck, Germany) containing 2.2 g of Span 80 (Sigma, USA) at 600 rpm at 40 °C for 10 min. Five grams of aqueous solution containing 1.7 g of Tween 80 (Merck, Germany) was then added and the mixture stirred for another 5 min. It was then cooled to 15 °C with the aid of an ice water bath and further stirring continued for 10 min to induce gelation of the dispersed droplets of gellan gum solution to form microspheres. This was followed by the addition of 20 g of 25.0%w/w calcium chloride dihydrate (Merck, Germany) solution, which was allowed to react for 15 min. The resultant mixture was finally left to stand in a shaker water bath to enable complete congelation of the gellan gum. The yeast-gellan gum microspheres, which constitute the micro-bioreactors, were harvested by filtration *in vacuo*. The microspheres were rinsed with 10 mL of sterile deionized water thrice and washed again with 100 mL of sterile deionized water to remove the iso-octane residue. Each batch of micro-bioreactors was prepared using the above procedure with the amount of materials stated. All the materials used, except gellan gum solution, were sterilized by filtration before use.

### 2.3. Study of Emulsification Process Effect on Yeast Viability

The microencapsulation process was simulated in this study. All materials and parameters used were identical, except for gellan gum, which was replaced with sterile deionized water in this study. Fifty grams of yeast suspension containing approximately 1 × 10^9^ cells without gellan gum was subjected to the emulsification process employed for the microencapsulation of yeast. The viability of the yeast cells at the end of the emulsification and after 2 h of standing in the shaker water bath were determined respectively by the spread plate method. Serial dilution of the samples was carried out using sterile distilled water. 100 μL of each dilution was spread onto malt extract agar (Oxoid, England) and incubated at 37 °C for 3 days. The number of viable yeast cells, represented by the number of colonies formed, was determined. Each experiment was carried out in triplicate.

### 2.4. Physical Characterization of Micro-Bioreactors

The morphology of the gellan gum microspheres with encapsulated yeast cells was determined using a light microscope (BX61-TRF, Olympus, Japan) connected to an image analyzer (DXC–390P 3CCD, Sony, Japan). The size and shape of 600 microspheres were evaluated using the Image Pro-Express 6.3 Software. The morphologies of the micro-bioreactors before and at the end of each fermentation cycle were compared.

### 2.5. Fermentation Using Micro-Bioreactors or Free Yeast

Fermentation was carried out using 30%w/w sucrose (SIS, Singapore) in malt extract broth (Oxoid, England) as the fermentation medium. All the micro-bioreactors harvested in each batch were introduced into 300 g of the fermentation medium in a conical flask. Fermentation was carried out under anaerobic condition using a loop trap setup, agitated in a shaker water bath at 90 strokes per min and 30 °C. Two-milliliter samples of the fermentation medium were withdrawn at specific time intervals for assay of ethanol. The fermentation study was repeated six times and the average results reported.

In a separate study, 2 and 3 batches of the micro-bioreactors were combined and subjected to fermentation respectively to investigate the effect of cell load on the fermentation efficiency. The viable counts of the liberated cells and ethanol yields of the micro-bioreactors were determined at different time intervals.

### 2.6. Assay of Ethanol

The ethanol produced was extracted from the fermentation medium using a procedure adapted from Offemen *et al.* [20,21]. The sample of fermentation medium collected was passed through a 0.45 μm membrane filter. One milliliter of 2-ethyl-1-hexanol (Synthesis Grade, Merck, Germany) was added to an equal volume of the filtrate. The mixture was agitated for 5 min using a vortex mixer (Fisons WhirliMixer, England). It was then centrifuged (RA-50J, Kubota 1720, Japan) at 3300 × *g* and 22 °C for 5 min to obtain complete phase separation. The upper phase was then separated and the above procedure was repeated twice. The upper phases collected were combined and assayed for ethanol by gas chromatography-mass spectrometry (GC-MS QP2010i, Shimadzu, Japan) with acetone (HPLC Grade, LabScan, Ireland) as the internal standard. Sample of 0.1 μL was injected into a BPWax 20 column (0.25 μm film thickness, 30 m × 0.25 mm I.D., SGE, USA). The injection port was maintained at 240 °C throughout the assay while the column was kept at 40 °C for 3 min, increased to 200 °C at a rate of 50 °C/min and then maintained at 200 °C for 10 min. The carrier gas, composed of purified helium was maintained at 51.3 kPa.

### 2.7. Viable Count of Free Yeast Cells in the Medium during Fermentation

Fermentation was carried out using the micro-bioreactors according to the method previously described. One-mL samples of the fermentation medium were withdrawn at different time intervals to determine the viable count of the free yeast cells by the spread plate method as mentioned previously. Each experiment was conducted in triplicate. Preliminary study showed that the yeast cells encapsulated in the microspheres did not develop into colonies in the spread plate method.

### 2.8. Fermentation Using Recycled Micro-Bioreactors

The micro-bioreactors were recovered from the fermentation medium by filtration *in vacuo* after 12 days of fermentation and then introduced into a fresh medium for fermentation as described previously. Two-milliliter samples of the fermentation medium from the second cycle were taken at specific time intervals for assay of ethanol. The fermentation cycle was carried out repeatedly for 10 cycles and the ethanol yield at the end of each cycle was determined.

### 2.9. Statistical Analysis

Data were reported as mean ± standard deviation (SD). Differences were assessed for significance using the unpaired *t*-tests with level of significance set at α = 0.05.

## 3. Results

### 3.1. Yeast-Gellan Gum Micro-Bioreactors

**Figure 1.**
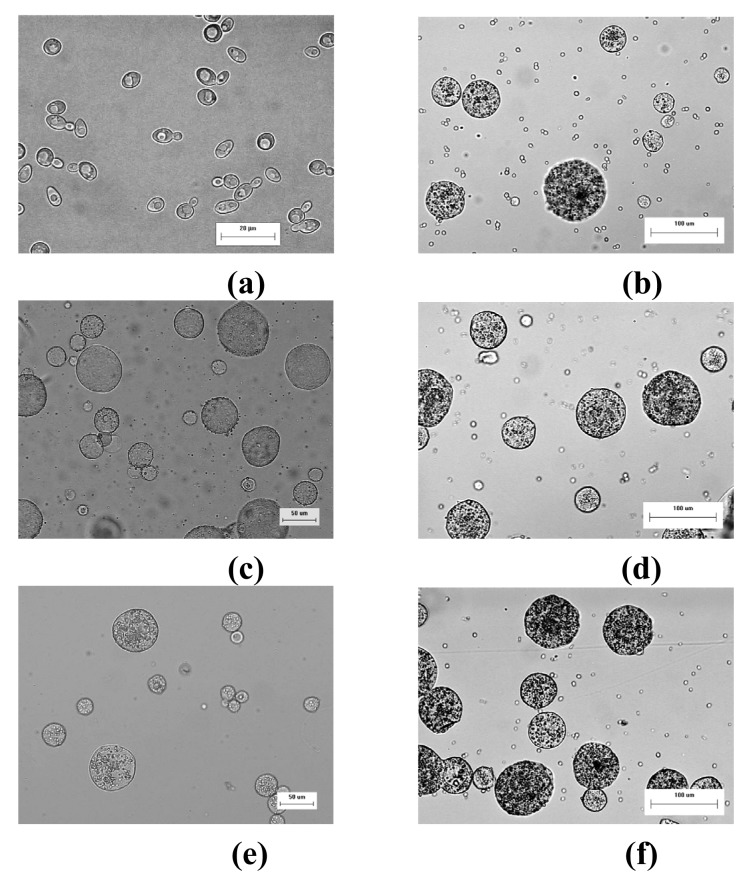
Photographs of (a) free yeast cells, (b) blank gellan gum microspheres, (c) encapsulated yeast cells in gellan gum microspheres and re-used yeast-gellan gum micro-bioreactors after the (d) first, (e) second and (f) tenth fermentation cycles.

Yeast cells were successfully encapsulated in gellan gum microspheres to form micro-bioreactors using the emulsification method (Figure 1a–c). Unencapsulated yeast cells were not detected by microscopic examination of the freshly harvested samples of micro-bioreactors. It appeared that all yeast cells were successfully encapsulated within the gellan gum microspheres. The microspheres, with cells scattered within the matrix (Figure 1c), were similar to blank microspheres in physical dimensions (Figure 1b). The micro-bioreactors produced were relatively discrete and spherical, with mean diameter of 36.7 (± 5.6) μm.

### 3.2. Viability of Yeast Cells Subjected to the Emulsification Process Employed in Microencapsulation

The viable count of the yeast was found to reduce from 10^9^ to 10^8^ cells after the emulsification process (Table 1). A slight decrease in cell viability was observed after standing in the reaction mixture for 2 h. The yeast cells recovered also required additional two days of incubation to form colonies on the agar plates.

### 3.3. Fermentation Efficiency of Yeast-Gellan Gum Micro-Bioreactors

**Figure 2.**
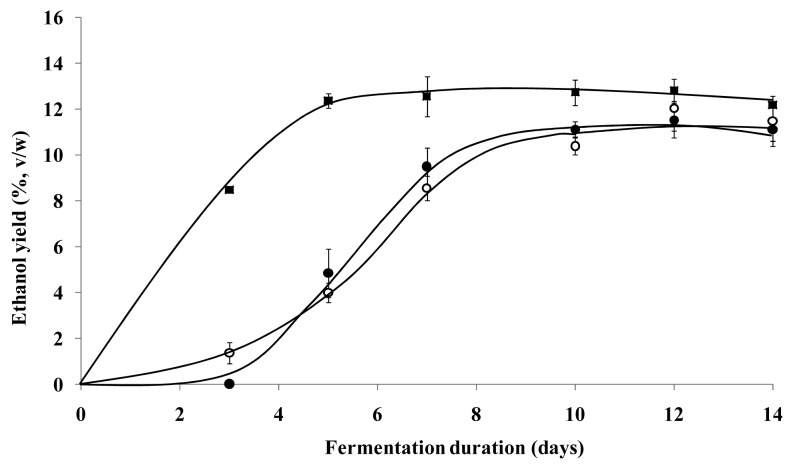
Fermentation efficiency of free yeast cells (■) and yeast-gellan gum micro-bioreactors in the first (●) and second (○) fermentation cycles.

**Figure 3.**
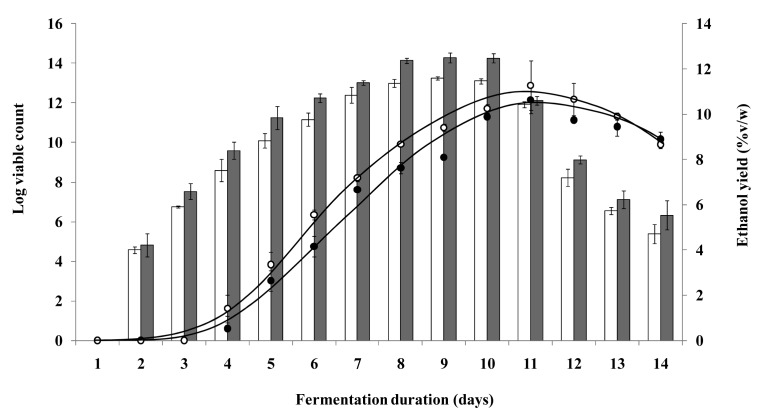
Viable count of free cells (bar) and fermentation efficiency (line) of two batches ( 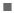, ●) and three batches (□, ○) of yeast-gellan gum micro-bioreactors

The maximum ethanol yields produced by both the free yeast and the micro-bioreactors were 12.8 (± 1.2)%v/w and 11.5 (± 0.8)%v/w while the time taken was about 5 and 12 days, respectively (Figure 2). The maximum ethanol yield of 12.0%v/w produced by the micro-bioreactors in the second fermentation cycle was observed after 12 days of fermentation (Figure 3). The pH value of the fermentation medium using the free yeast or the micro-bioreactors was reduced from 4.7 before the fermentation process to approximately 3.4 after 14 days of fermentation.

### 3.4. Changes in Yeast-Gellan Gum Micro-Bioreactors after Fermentation

The micro-bioreactors were retrieved at the end of the fermentation process for further investigation. When observed under the light microscope, the micro-bioreactors were found to remain intact and spherical despite being immersed in the fermentation medium over a prolonged duration of 14 days, indicating that the gellan gum matrix was relatively strong (Figure 1d). However, an appreciable number of free yeast cells was observed in the fermentation medium (Figures 1d–f).

### 3.5. Viable Count of Free Yeast Cells in the Fermentation Medium

The viable count of free yeast cells in the fermentation medium containing micro-bioreactors was determined after 5 and 12 days respectively to assess the extent of cell breakthrough from the micro-bioreactors. The viable cell count of the fermentation medium to which an equivalent amount of free yeast was added was similarly determined. The time intervals chosen corresponded to the maximum ethanol yields of the free yeast and micro-bioreactors. The viable count of yeast cells present in the media during fermentation using free yeast (FY) and micro-bioreactors (MY) respectively were determined (Table 2).

### 3.6. Fermentation Efficiency and Viable Count of Yeast Cells Liberated into the Medium during Fermentation Using Two and Three Batches of Micro-Bioreactors

The effect of different loads of micro-bioreactors on fermentation efficiency was investigated in an attempt to elucidate the contribution of the free yeast cells and the micro-bioreactors to the production of ethanol. The fermentation profiles of the different batch sizes were similar (Figure 3). The viable counts of liberated cells from the different batch sizes of micro-bioreactors increased, leveled off and decreased over time (Figure 3). Highest viable counts and ethanol yields (approximately 11%v/w) were observed at about 11 days regardless of the micro-bioreactor batch size. At the maximum ethanol yield, the viable count of liberated yeast cells for the two or three combined batches (about 10^12^ viable cells) was higher than that for the single batch (10^7^ viable cells). Comparable ethanol yields were obtained despite the significant difference in viable count (*p* > 0.05).

### 3.7. Re-Usability and Stability of Yeast-Gellan Gum Micro-Bioreactors

**Figure 4.**
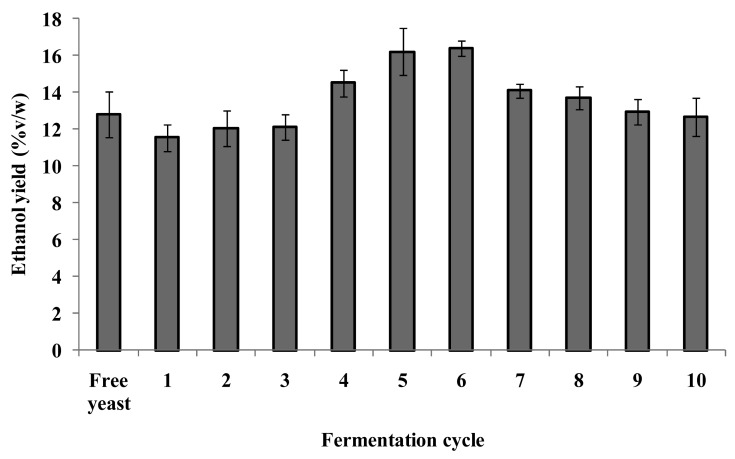
Fermentation efficiency of free yeast cells and re-used yeast-gellan gum micro-bioreactors.

The micro-bioreactors were recovered from the fermentation medium and gently rinsed on the filter paper. Most of the liberated free yeast cells would be washed away and the amount of free yeast cells retained on the micro-bioreactors was considered insignificant. As such, the free yeast cells in the fermentation medium were not recycled. The present study shows that the micro-bioreactors could be easily recovered by filtration and re-used up to 10 fermentation cycles with high fermentation efficiency (Figure 4). The appearance of the micro-bioreactors after a number of fermentation cycles is shown in Figures 1d-f. The micro-bioreactors remained intact and spherical even after 10 fermentation cycles, indicating that the gellan gum matrix remained strong in the fermentation medium.

## 4. Discussion

In the emulsification process, an organic solvent, surfactants, prolonged stirring at high speed and elevated temperature were employed. It was deemed important to investigate the summative effect of these factors on the viability of the yeast cells since this is of paramount importance. The gellan gum microspheres could only be broken down by employing high temperature or enzymes to liberate the yeast cells for viable count. Such conditions employed are likely to harm the yeast cells and result in lower viable count. As such, the effect of a simulated emulsification process using yeast cells in an aqueous medium without gellan gum was studied. One log reduction in cell viability was observed after the microencapsulation process. In the present study, the microspheres were allowed to stand in the reaction mixture for 2 h to further harden the microspheres. This step in the process was found to have insignificant effect (*p* > 0.05) on the viability of the yeast cells, as only a slight decrease in cell viability was observed after standing in the reaction mixture for 2 h. The overall results clearly showed that the emulsification process employed was feasible for encapsulating yeast cells to produce micro-bioreactors for fermentation. However, the yeast cells recovered required an additional two days of incubation to form colonies on the agar plates. This indicated that the emulsification process had affected cellular activities and repressed the yeast cell growth.

The fermentation efficiency of the micro-bioreactors was evaluated by determination of the quantity of ethanol produced during fermentation. All the harvested microspheres containing approximately 10^8^ viable yeast cells were introduced into the fermentation medium. An equivalent amount of free yeast was used to replace the micro-bioreactors as the control for this study. The ethanol yield for both free yeast and micro-bioreactors increased over time and then leveled off or decreased slightly (Figure 2). Reduction in cell viability and ethanol production due to nutrient deficiency or toxic effects of cumulative metabolites is well-established [22]. Since not all the sucrose used in the present study was fermented, the stationary phase of the ethanol production could be attributed to the inhibition of yeast by deficiency of growth factors in the malt extract medium and/or the adverse changes to the environmental conditions. The slight decrease in ethanol yield was probably due to oxidation of the ethanol to ethanoic acid or loss of ethanol by evaporation over a prolonged period of time. The pH value of the fermentation medium using the free yeast or the micro-bioreactors was reduced from 4.7 to approximately 3.4 after 14 days of fermentation. The decrease in pH of the fermentation medium could be attributed to the accumulation of ethanoic acid and carbon dioxide produced during fermentation.

The difference in maximum ethanol production by the encapsulated yeast cells within the micro-bioreactors and the free yeast cells was found to be insignificant (*p* > 0.05). A lag phase in ethanol production was observed in the first three days of the first fermentation cycle using the micro-bioreactors (Figure 2). This phenomenon could be attributed to the stress induced on the yeast cells by the encapsulation process. As mentioned previously, the yeast cells subjected to a simulated emulsification process took a longer incubation time to form colonies, indicating that the metabolism of the yeast cells was affected by the process. Several studies had reported that the metabolic, physiological and morphological properties of yeast cells were affected after the encapsulation process [23–25]. Restriction in the transfer of nutrients, ethanol and other metabolites due to the matrix barrier of the microspheres could also have contributed to the lag phase observed [6]. The time required for the nutrient supply to reach the yeast cells entrapped in the core of the microspheres was more prolonged, thus the growth and fermentation ability of the encapsulated could be repressed.

The fermentation curve of the micro-bioreactors in the second fermentation cycle was found to be different from the first fermentation cycle (Figure 2). The lag phase present in the first fermentation cycle was not observed in the second fermentation cycle. The absence of the lag phase indicates that the encapsulated yeast cells were indeed affected by the stress induced during the encapsulation process, suppressing the cellular activity of the yeast cells as mentioned earlier. Over time, the encapsulated yeast cells convalesced from the stress and were able to perform fermentation more efficiently. However, similar to the first fermentation cycle, the micro-bioreactors still required 12 days of fermentation to attain the maximum ethanol yield of 12%v/w despite the absence of the lag phase. The ethanol yield of the second fermentation cycle was comparable to that of the free yeast (12.8%v/w) (*p* > 0.05). This seems to suggest that the longer fermentation time required by the micro-bioreactors was largely attributed to the presence of the gellan gum matrix barrier restricting the mass transfer of nutrients and metabolites, thereby affecting the cellular activities and performance of the encapsulated yeast cells.

The encapsulated cells at the periphery of the microspheres had produced buds that detached themselves from the microspheres. Koyama and Seki [1] reported that a high concentration of yeast cells below the surface of a polymer matrix might weaken the matrix, thereby enabling detachment of the yeast cells from the surface. Inama *et al.* [26] reported that prolonged contact between entrapped yeast cells and sucrose led to cell proliferation and detachment of the yeast buds into the fermentation medium. The carbon dioxide released during fermentation by the encapsulated yeast cells might also have contributed to some deformation of the polymer matrix and facilitated cell release into the fermentation medium [6]. The growth of yeast cells has been reported to be associated with the magnitude of the polymer matrix porosity [27]. Polymer matrix with large pore size is desirable to promote the growth of encapsulated yeast cells as well as the mass transfer of nutrients and metabolites to achieve high ethanol yield [26,27]. While highly porous encapsulant matrices favor cellular activities, they allow breakthrough of yeast cells. The latter is undesirable because the detached yeast cells will no longer be protected from environmental stress and ethanol toxicity and their viability will be compromised. Nevertheless, the ability of the gellan gum matrix to remain intact in the fermentation medium clearly indicated its usefulness as an encapsulant matrix for the production of micro-bioreactors that can be employed in continuous fermentation processes.

Table 2 shows the corresponding ethanol yield of both the free yeast and the micro-bioreactors at the respective fermentation time. The viable count for FY increased markedly from 10^8^ to 10^13^ cells after 5 days of fermentation, yielding 12.4%v/w ethanol. The viable count decreased markedly to 10^7^ cells after 12 days but there was insignificant change in the ethanol yield (*p* > 0.05), indicating the harmful effect of prolonged exposure to about 12%v/w ethanol. On the other hand, the viable count of free yeast cells in the fermentation medium for MY after 5 days was high (about 10^9^ cells), indicating significant cell breakthroughs from the microspheres, probably followed by cell replication in the medium. However, the corresponding ethanol yield was relatively low (4.9%v/w). Collectively, the results confirmed the earlier postulation that the cellular activities and fermentation ability of the yeast was transiently affected by the encapsulation process. After 12 days, which corresponded to the time taken by the micro-bioreactors to attain maximum ethanol production, the viable count decreased to 10^7^ while the ethanol yield increased markedly. The significance of ethanol toxicity was further illustrated by the relatively similar viable counts (about 10^7^ cells) at ethanol yield of about 12%v/w for FY and MY after 12 days. The maximum concentration of ethanol produced by the free yeast was comparable to that produced by the micro-bioreactors (11.5%v/w) (*p* > 0.05), suggesting that the free yeast cells played a greater role in fermentation. The micro-bioreactors could also carry out fermentation but they appeared to play a more important role as a reservoir to generate free yeast cells into the medium to carry out fermentation concurrently. The free yeast cells were not protected from the ethanol toxicity, which aptly explained the decrease in viable count.

It was observed that the amount of liberated cells was not directly proportional with the micro-bioreactors batch size and the fermentation efficiency of the yeast was affected by the concentration of ethanol produced in the fermentation medium (Figure 3 and Table 2). The micro-bioreactors of different batch sizes took about 4 days to yield 10^8^ liberated yeast cells and another 7 days to achieve maximum ethanol yield. It was also observed that the ethanol yield increased with increase in viable count of liberated cells over time (Figure 3). Collectively, the results clearly showed that the liberated yeast cells played a greater role than the encapsulated yeast cells in the production of ethanol. The fermentation activity of the liberated yeast cells were inhibited by the high ethanol concentration in the fermentation medium. Hence, the maximum ethanol yields did not increase beyond 12%v/w despite the increase in amount of micro-bioreactors used. Besides contributing to the production of ethanol, the micro-bioreactors played a greater role as a reservoir for generation of free yeast cells to carry out fermentation.

Compared to the free yeast (control), the micro-bioreactors were able to perform fermentation efficiently when re-used in subsequent fermentation processes (Figure 4). There was a gradual increase in ethanol yield in the first six cycles. The ethanol yield of the first fermentation cycle was 11.5%v/w, which was comparable to that of the free yeast (12.8%v/w) (*p* > 0.05). The ethanol yield gradually increased after the first fermentation cycle to reach the maximum of 16.4%v/w in the sixth fermentation cycle, which was significantly higher than that of the free yeast (*p* < 0.05). As discussed previously, the contribution of the free yeast cells to the production of ethanol was relatively constant. It could therefore be inferred that the contribution of the encapsulated yeast cells to ethanol production increased over time. This could be attributed to the increase in yeast cell density in the micro-bioreactors [28]. Due to degradation, the gellan gum matrix became sufficiently porous and enabled more efficient mass transfer, thereby enhancing yeast budding and allowing fermentation to take place. The gellan gum matrix was able to protect the encapsulated yeast from the growth inhibitory effects of environmental stress and ethanol. Due to concentration gradient, the ethanol produced by the encapsulated yeast cells would diffuse out of the micro-bioreactors into the fermentation medium. Moreover, contact between the ethanol in the fermentation medium and the encapsulated yeast would be reduced to a certain extent by the gellan gum matrix that surrounded the cells. It is important to bear in mind that this would similarly affect the accessibility of nutrients to the cells. Hence, the ethanol yield would be determined by the intricate balance between the aforementioned effects. A decrease in fermentation performance was observed after the sixth fermentation cycle. The ethanol yield decreased and leveled off to 12.9%v/w in the ninth fermentation cycle. Despite the fall, the ethanol yield was still comparable to that of the free yeast (*p* > 0.05). The decrease might be associated with the weakening of the gellan gum matrix, compromising its protective function.

Compared to free yeast cells, the fermentation time required by the micro-bioreactors in batch processes was longer as time was needed for liberation of yeast cells into the fermentation medium to carry out fermentation. However, the micro-bioreactors have the important advantage of potential application in continuous fermentation. The disadvantage of longer fermentation time would be avoided in a continuous fermentation process where a relatively constant number of free yeast cells is sustained. The micro-bioreactors can be used continuously in the fermentation vessel with continuous supply of substrate into the vessel. In contrast, batch fermentation processes using free yeast cells would incur additional cost and time to revive a fresh yeast culture as well as cleaning and sterilization of the fermentation system. In addition, the viability and fermentation efficiency of the free yeast cells had been reported to be markedly decreased by their long exposure to ethanol during fermentation, rendering them unsuitable for re-use [22]. Furthermore, free yeast cells can only be recovered from the fermentation medium by centrifugation, which is not economical for industrial application. Conversely, the yeast-gellan gum micro-bioreactors can be easily recovered from the fermentation medium by filtration. Moreover, the micro-bioreactors could be packed in a perforated column setup to facilitate separation of the micro-bioreactors from the solid feedstock.

## 5. Conclusions

Yeast cells were successfully encapsulated in gellan gum microspheres using the emulsification method. The viability of the yeast was not markedly affected by the conditions employed in the encapsulation process. Relatively discrete and spherical micro-bioreactors could be obtained. The ethanol production in the first three cycles using micro-bioreactors was comparable to that using free yeast. The micro-bioreactors were also easily recovered from the fermentation medium by filtration and could be re-used for at least 10 fermentation cycles with relatively high ethanol yields. The micro-bioreactors were stable and strong, remaining intact throughout repeated use. The high stability of the micro-bioreactors during fermentation is very important, especially for industrial application involving continuous fermentation processes. Over time, the micro-bioreactors became sufficiently porous and enabled efficient mass transfer. Although they allowed cell breakthrough, they also enabled proliferation of the encapsulated cells within the micro-bioreactors. Overall, gellan gum is a useful encapsulant polymer for yeast cells to produce re-usable micro-bioreactors for continuous fermentation to produce bio-ethanol.

## Figures and Tables

**Table 1.** Viability of yeast cells subjected to the emulsification process.

	**Viable count, n**	**Log_10_ n**	**Percent viability**
Before emulsification	1.83 (± 0.44) × 10^9^	9.26	100.00
After emulsification	4.50 (± 0.55) × 10^8^	8.65	93.41
After 2 hours of standing in the reaction mixture	3.46 (± 0.15) × 10^8^	8.54	92.22

**Table 2.** Viable counts of free yeast cells in the fermentation media and their corresponding ethanol yields.

**Type of fermentor**	**Fermentation duration (days)**	**Number of cells (n) found in the media**	**log_10_ n**	**Ethanol yield (%v/w)**
Free yeast	**0**	1.30 (± 0.23) × 10^8^	8.11	0
	**5**	5.51 (± 1.01) × 10^13^	13.74	12.4
	**12**	5.01 (± 2.10) × 10^7^	7.70	12.8

Micro-bioreactors (encapsulated yeast)	**0**	0	0	0
	**5**	7.52 (± 0.70) × 10^9^	9.88	4.9
	**12**	6.91 (± 0.38) × 10^7^	7.84	11.5

## References

[1] Koyama K., Seki M. (2004). Cultivation of yeast and plant cells entrapped in the low-viscous liquid-core of an alginate membrane capsule prepared using polyethylene glycol. J. Biosci. Bioeng..

[2] Melzoch K., Rychtera M., Habova V. (1994). Effect of immobilization upon the properties and behavior of *Saccharomyces cerevisiae* cells. J. Biotechnol..

[3] Dror Y., Cohen O., Freeman A. (1988). Stabilization effects on immobilised yeast: Effect of gel composition on tolerance to water miscible solvents. Enzyme Microb. Technol..

[4] Holcberg I.B., Margalith P. (1981). Alcoholic fermentation by immobilized yeast at high sugar concentrations. Appl. Microbiol. Biotechnol..

[5] Barron N., Brady D., Love G., Marchant R., Nigam P., McHale L., McHale A.P., Wijffels R.H., Buitelaar R.M., Bucke C., Tramper J. (1996). Alginate-immobilised thermotolerant yeast for conversion of cellulose to ethanol. Immobilized Cells: Basics and Applications. International Symposium on Applied Biocatalysis.

[6] Raymond M., Neufeld R.J., Poncelet D. (2004). Encapsulation of brewers yeast in chitosan coated carrageenan microspheres by emulsification/thermal gelation. Artif. Cells Blood Substit. Immobil. Biotechnol..

[7] Roukas T. (1996). Ethanol production from non-sterile beet molasses by free and immobilised *Saccharomyces cerevisiae* cells using fed-batch culture. J. Food Eng..

[8] Sun Z.J., Lv G.-J., Li S.-Y., Yu W.-T., Wang W., Xie Y.-B., Ma X. (2007). Differential role of microenvironment in microencapsulation for improved cell tolerance to stress. Appl. Microbial. Cell Physiol..

[9] Nedović V.A., Obradović B., Leskošek-Čukalović I., Trifunović O., Pešić R., Bugarski B. (2001). Electrostatic generation of alginate microbeads loaded with brewing yeast. Process Biochem..

[10] Stormo K.E., Crawford R.L. (1992). Preparation of encapsulated microbial cells for environmental applications. Appl. Environ. Microbiol..

[11] Chan L.W., Heng P.W.S., Wan L.S.C. (1997). Effect of cellulose derivatives on alginate microspheres prepared by emulsification. J. Microencapsul..

[12] Chan L.W., Liu X., Heng P.W.S. (2005). Liquid phase coating to produce controlled-release alginate microspheres. J. Microencapsul..

[13] Heng P.W.S., Chan L.W., Wong T.W. (2003). Formation of alginate microspheres produced using emulsification technique. J. Microencapsul..

[14] Wan L.S.C., Heng P.W.S., Chan L.W. (1992). Drug encapsulation in alginate microspheres by emulsification. J. Microencapsul..

[15] Wong T.W., Chan L.W., Lee H.Y., Heng P.W.S. (2002). Release characteristics of pectin microspheres prepared by an emulsification technique. J. Microencapsul..

[16] Jansson P.E., Lindberg B., Sandford P.A. (1983). Structural studies of gellan gum, an extracellular polysaccharide elaborated by *Pseudomonas elodea*. Carbohydr. Res..

[17] Milas M., Shi X., Rinaudo M. (1990). On the physicochemical properties of gellan gum. Biopolymers.

[18] O'Neill M.A., Selvendran R.R., Morris V.J. (1983). Structure of the acidic extracellular gelling polysaccharide produced by *Pseudomonas elodea*. Carbohydr. Res..

[19] Yuguchi Y., Urakawa H., Kajiwara K. (2002). The effect of potassium salt on the structural characteristics of gellan gum gel. Food Hydr..

[20] Offemen R.D., Stephenson S.K., Robertson G.H., Orts W.J. (2005). Solvent extraction of ethanol from aqueous solutions. I. Screening methodology for solvents. Ind. Eng. Chem. Res..

[21] Offemen R.D., Stephenson S.K., Robertson G.H., Orts W.J. (2005). Solvent extraction of ethanol from aqueous solutions. II. Linear, branched and ring-containing alcohol solvents. Ind. Eng. Chem. Res..

[22] Desimone M.F., Degrossi J., Aquino M.D., Diaz L.E. (2002). Ethanol tolerance in free and sol-gel immobilised *Saccharomyces cerevisiae*. Biotechnol. Lett..

[23] Doran P.M., Bailey J.E. (1985). Effects of immobilisation on growth, fermentation properties, and macromolecular composition of *Saccharomyces cerevisiae* attached to gelatin. Biotechnol. Bioeng..

[24] Hilge-Rotmann B., Rehm H. (1990). Comparison of fermentation properties and specific enzyme activities of free and calcium-alginate-entrapped *Saccharomyces cerevisiae*. Appl. Microbiol. Biotechnol..

[25] Talebnia F., Taherzadeh M.J. (2007). Physiological and morphological study of encapsulated *Saccharomyces cerevisiae*. Enzyme Microb. Technol..

[26] Inama L., Diré S., Carturan G., Cavazza A. (1993). Entrapment of viable microorganisms by SiO_2_ sol-gel layers on glass surfaces: Trapping, catalytic performance and immobilisation durability of *Saccharomyces cerevisiae*. J. Biotechnol..

[27] Kumakura M., Yoshida M., Asano M. (1992). Preparation of immobilised yeast cells with porous substrates. Process Biochem..

[28] Mei L.H., Yao S.J. (2002). Cultivation and modeling of encapsulated *Saccharomyces cerevisiae* in NaCS-PDMDAAC polyelectrolyte complexes. J. Microencapsul..

